# Effects of Land cover change on Great Apes distribution at the Lobéké National Park and its surrounding Forest Management Units, South-East Cameroon. A 13 year time series analysis

**DOI:** 10.1038/s41598-018-36225-2

**Published:** 2019-02-05

**Authors:** Yisa Ginath Yuh, Zacharie N. Dongmo, Paul K. N’Goran, Herbert Ekodeck, Achile Mengamenya, Hjalmar Kuehl, Tenekwetche Sop, Wiktor Tracz, Michael Agunbiade, Tangwa Elvis

**Affiliations:** 1 Warsaw University of Life Sciences (WULS-SGGW), Nowoursynowska 161, 02-787 Warszawa, Poland; 2 Eberwalde University for Sustainable Development (HNEE), Schicklerstraße 5, 16225 Eberswalde, Germany; 3 Max Planck Institute for Evolutionary Anthropology (MPI EVAN), Deutscher Platz 6, 04013 Leipzig, Germany; 4 World Wide Fund for Nature (WWF), Cameroon Country Program Office, Rue La Citronelle, BAT Compound Bastos, P.O. Box 6776, Yaoundé, Cameroon; 5 World Wide Fund for Nature (WWF), Regional Office for Africa – Yaoundé Hub, Rue La Citronelle, BAT Compound Bastos, P.O. Box 6776, Yaoundé, Cameroon; 6 German Centre for Integrative Biodiversity Research (iDiv) Halle-Leipzig-Jena, Deutscher Platz 5e, 04103 Leipzig, Germany; 7 Ministère des Forêts et de la Faune, Parc National de Lobéké, Po Box 15, Yokadouma, Cameroon

## Abstract

Understanding the effects of land cover change on wildlife distribution is very important for resource management and conservation planning. This paper aimed at detecting the effects of land cover change on great apes distribution at the Lobéké National Park and its bounded forest management units (FMUs). Data on great ape nests were collected in the field for the years 2001 and 2014 through distance sampling with line transects. Landsat TM images of South-East Cameroon for the years 2001 and 2014 were acquired from earth explorer and corrected atmospherically for proper visualization. An area of interest comprising the Lobéké National Park and its FMUs was extracted for classification and change detection. A comparison in great apes nest distribution and change per land cover change category was done for both years through point pattern analysis, whereas a time series analysis of the detected land cover change impacts on great apes nest distribution for a period of 13 years was modeled using logistic growth and regression equations in Vensim 7.2. The results could illustrate that, as land cover changes from one cover type in 2001 to another in 2014, increases or decreases in great apes nests were observed within each changed area.

## Introduction

Great Apes are large mammal species originating from the Order: “Primates” and Family: “Hominoidea”, and are the closest relatives to humans. These species include chimpanzees, gorillas and bonobos inhabiting several habitat types across Africa, as well as orangutans, inhabiting South-East Asian forests. In order to plan and ensure appropriate protection and sustainable management of the said endangered species including other species under similar threats, it is important to understand and obtain concrete data on their range and distribution, as well as density and habitat diversity^1^. With such data, a continuous monitoring plan can be developed in order to mitigate the effects of extinction and secure species niche^2^.

In Cameroon, two species of great apes exist namely: chimpanzees and gorillas. Chimpanzees are of two subspecies: *Pan troglodytes troglodytes* and *Pan troglodytes ellioti*. The former is distributed along forested areas in Southern Cameroon while the latter is confined to Central and West Cameroon, North of river sanaga. *Pan troglodyte ellioti* ranges from the Banyang-Mbo Wildlife Sanctuary with population estimates of approximately 500–900 individuals^3^ to the Ebo National park with estimates of about 626–1480 individuals, and to the Mbam and Djerem National Park with estimates of about 500 individuals^4^. The Western Gorilla (*Gorilla gorilla*) on the other hand has two identified subspecies: the Western lowland Gorilla (*Gorilla gorilla gorilla*) and the cross river Gorilla (*Gorilla gorilla diehli*)^5,6^. Cross-river gorillas are the main subspecies of Western gorillas inhabiting various forest areas within Cameroon and Nigeria with few species discovered in the Ebo forest in Cameroon. The taxonomic status of the discovered species is still to be clarified by researchers since little has been done to examine their distribution and abundance^7–9^. Studies have also revealed that approximately 250–300 cross river gorillas inhabit forest areas of about 12,000 square kilometers^10,11^ and are separated from the nearest population of Western lowland Gorillas by approximately 300 km^2^.

According to the IUCN species red list, the great ape species of Cameroon are classified as ‘critically endangered’ and of global conservation concern. Numbers are declining because of hunting for bush meat, disease and habitat fragmentation^12,13^ leading to the isolation of small populations which are likely to become genetically unviable in the long term. Much of the variation in species’ extinction risk is associated with spatial patterns of human threats and depends on how different species respond to threats^14,15^. These species occur at a very low density of less than 1 individual/km² of forest on average across much of their range and has a relatively slow reproductive rate, with one infant born every 4–5 years. As such, they considerably require extensive vegetation covers in order to survive. Alterations in their habitats as well as the habitats of other wildlife species are greatly influenced by humans. These human induced influences has caused huge changes in land use and land cover patterns which are now gaining recognitions as important drivers of environmental change^16^. In Cameroon as well as in other African countries, the driving forces or causes of land use and land cover change are connected to deforestation, subsistence farming with poor land use practices, mining, infrastructural development and rapid urbanization which intend alters the habitats of most wildlife species and as a consequence, contributes immensely to the devastating effects of climate change. Deforestation has been observed to be caused by high demands for forest wood, high demands for domestic fuel wood and increase demand for medicinal plants. These causes are highly attributed to illegal logging with an estimated 80% of Africa’s forest cover being lost in the early 2000s^17^, thus posing a negative impact on chimpanzee and gorilla densities and distribution^18^ as well as the distribution of other wildlife species. Subsistence farming on the other hand is linked with increase demand for agricultural products due to increase human population, while rapid urbanization and infrastructural development are also caused by the continuous growth in the continent’s population. These human induced problems are constantly causing serious threats to protected areas at large thus causing loss in wildlife corridors which may in turn lead to genetic drifting and inbreeding of species while negatively impacting population stability and distribution and to a greater extent loss in ecological integrity, extinction, as well as increase in human-wildlife conflict^19,20^. Due to such conflicts, frustration and animosity may occur between wildlife species, thus resulting in retaliatory killings^21,22^.

The main aim of the study was therefore to detect and analyze the effects of land cover changes to the distribution of great ape (Chimpanzee and Gorilla) species that has occurred at the Lobéké National Park and its surrounding forest management units (South-East Cameroon) within the course of 13 years (2001–2014). In order to attain this aim,

- We classified and analyzed the changes in Land cover types that have occurred at the study areas within the course of 13 years (2001–2014).
- We analyzed and compared the changes in occurrence and distribution of Chimpanzees and gorillas within the identified land cover classes of both years using nest count survey data. The comparison took into consideration, change effects within the National Park and its bounded forest management units (FMUs).
- We applied a time series modeling and analysis of changes in chimpanzee and gorilla nest distribution impacted by changing land cover types at a period of 13 years.

With the introduction of the Landsat program in the early 1970s, satellite remote sensing has been widely used as the best approach to manipulate and analyze satellite imageries at different scales. This approach in addition to GIS techniques offer an array of tools that facilitate data processing, data analysis and classification of earth surface features such as forest cover, soils, water bodies, landscapes, infrastructures, agricultural areas etc. to produce thematic maps^23–26^.

## Results

### Image classification, Great Apes distribution and change detection

The land cover classification results produced five categories of land cover types: roads and deforested patches, dense forest, swampy forest, rivers and grassland and low vegetation. These classification data were validated with a confusion matrix using a pivot table (Tables 1 and 2). The validated results indicated an overall classification accuracy of 90.5% in 2001 and 89% in 2014. A further validation was done through comparison in forest loss-forest gain calculations with global forest cover data. From the classification results as such, about 2.5% forest were lost in the park as compared to 4.7% in the FMUs. Meanwhile, 1.2% forests were gained in the park as compared to 2.9% gained in the FMUs. This amounts to an average forest loss of approximately 4% and gain of approximately 2% in the entire study area (Lobéké National Park + FMUs). Estimates from global forest data show approximately 6% forest loss and 1% forest gained in the entire study area, hence a closer results to validate our land cover classification (https://www.globalforestwatch.org/map/11/1.73/15.93/ALL/grayscale/loss,forestgain,forest2000?tab=analysistab&geostore=9d35e68a2a51930d61d2218ad907faca&begin=2001-01-01&end=2015-01-01&threshold=20&dont_analyze=true). Forest loss was calculated by summing up all land cover areas that have changed from forest to other land cover types in Table 5 (i.e. Dernse forest ->Grassland and low vegetation + Dernse forest ->River + Dernse forest ->Roads and deforested patches + Swampy forest ->Grassland and low vegetation + Swampy forest ->River + Swampy forest ->Roads and deforested patches). Meanwhile forest gain involved summing up all land cover types that have changed to forest (Grassland and low vegetation ->Dense forest + Grassland and low vegetation ->Swampy forest + Roads and deforested patches ->Dense forest + Roads and deforested patches ->Swampy forest). From the forest loss-gain estimates as well as the entire forest cover data in the study area, an approximated 93% in-tacked or unlogged forest exist in the entire study area.

**Table 1:** Accuracy assessment of classified land cover categories (2001).

Classification 2001	Dense forest	Roads and deforested patches	River	Grassland and low vegetation	Swampy forest	Total
Dense forest	28	0	0	0	3	31
Roads and deforested patches	0	21	0	0	8	29
River	5	0	6	0	0	11
Grassland and low vegetation	0	0	0	14	0	14
Swampy forest	0	0	0	0	23	23
Grand Total	33	21	6	14	34	**108**

Overall accuracy = 90.5%.

**Table 2:** Accuracy assessment of classified land cover categories (2014).

Classification 2014	Dense forest	Roads and deforested patches	River	Grassland and low vegetation	Swampy forest	Total
Dense forest	55	6	0	0	4	65
Roads and deforested patches	3	35	0	0	4	42
River	0	0	18	0	0	18
Grassland and low vegetation	8	0	0	10	2	20
Swampy forest	0	0	0	0	32	32
Grand Total	66	41	18	10	42	**177**

Overall accuracy = 89%.

**Figure 1:**
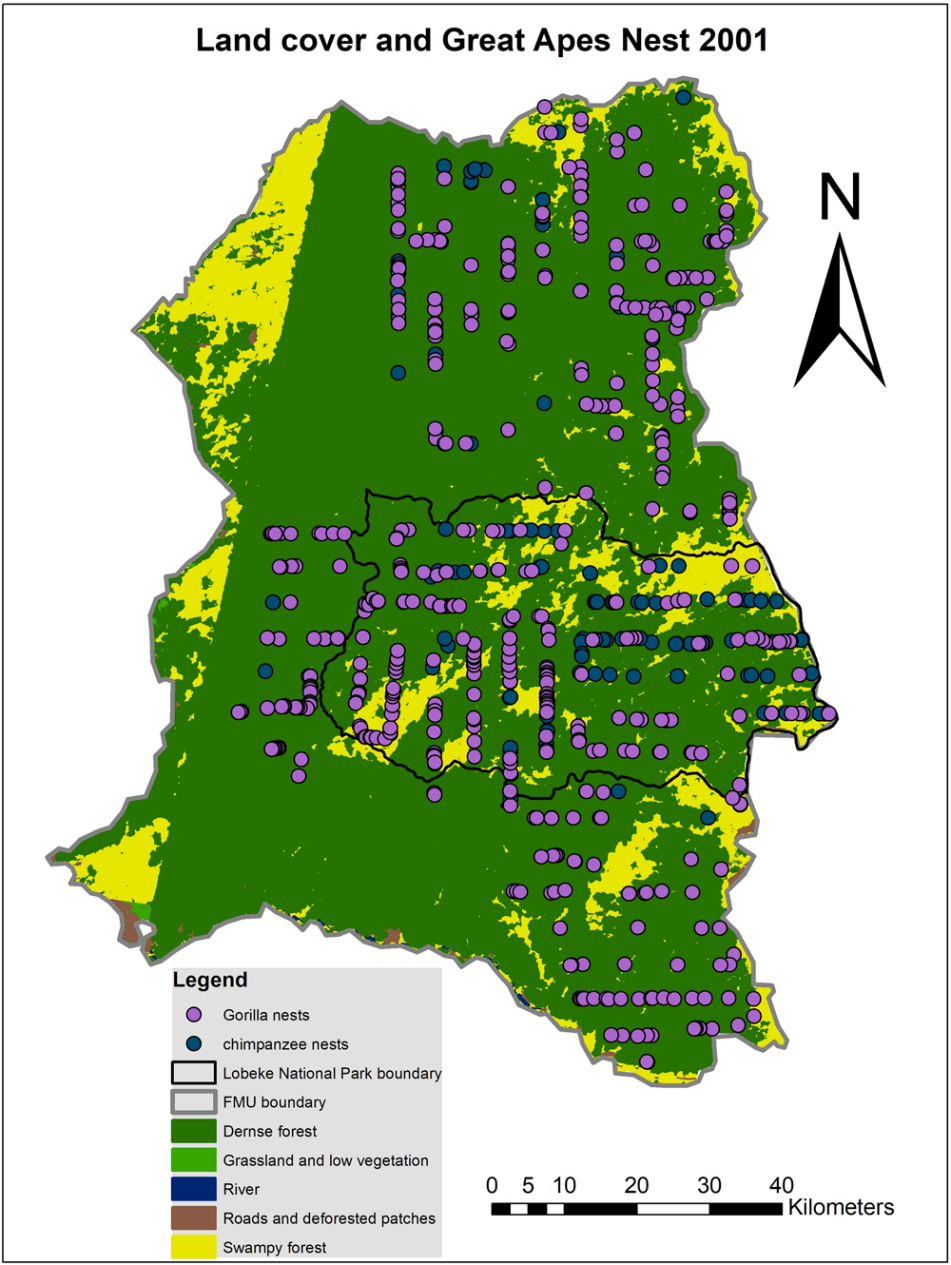
Pattern distributions in chimpanzee and gorilla nests within classified land cover categories for the year 2001(Map prepared with ArcGIS 10.3.1).

**Figure 2:**
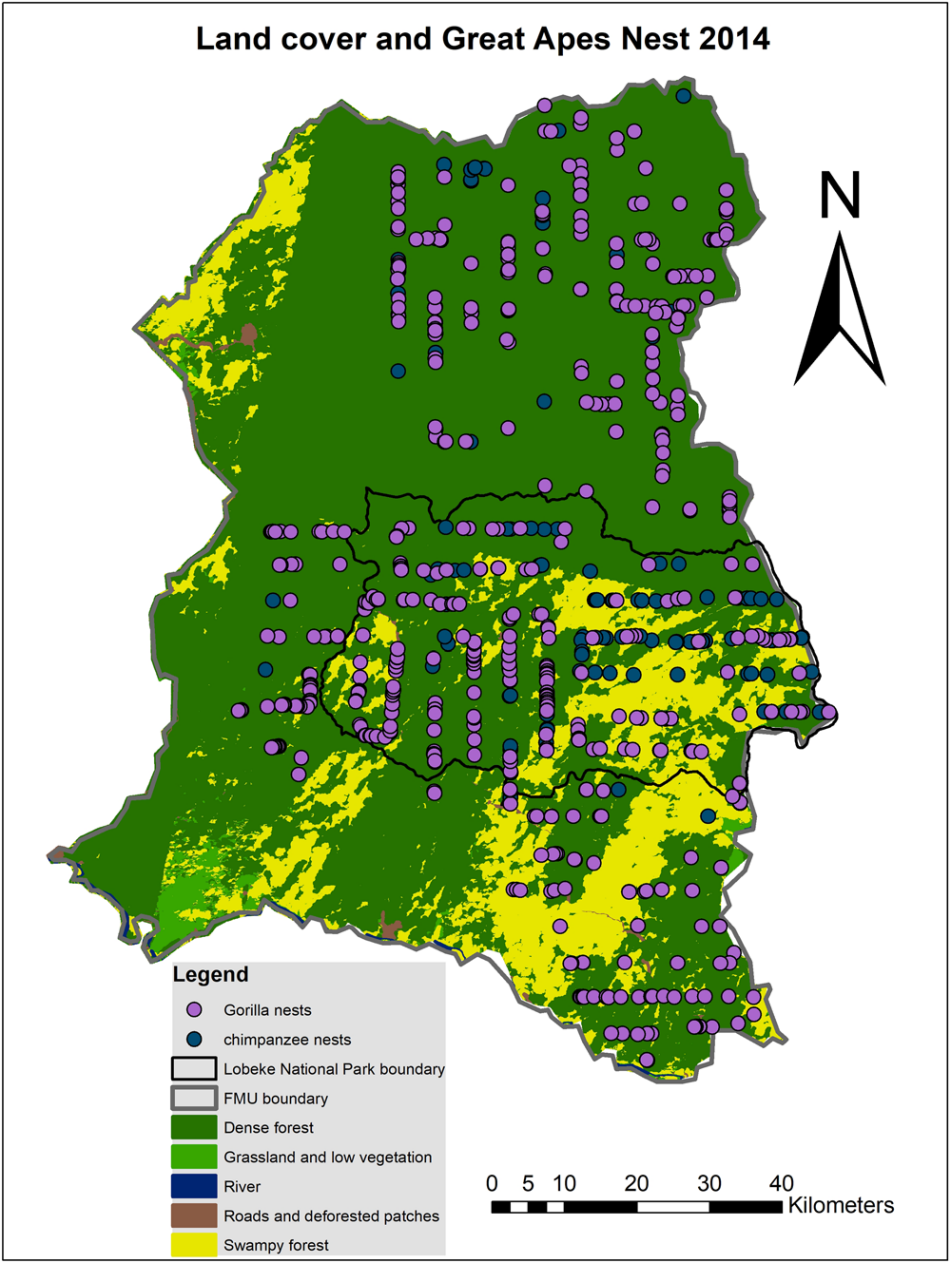
Pattern distributions in chimpanzee and gorilla nests within classified land cover categories for the year 2014(Map prepared with ArcGIS 10.3.1).

**Figure 3:**
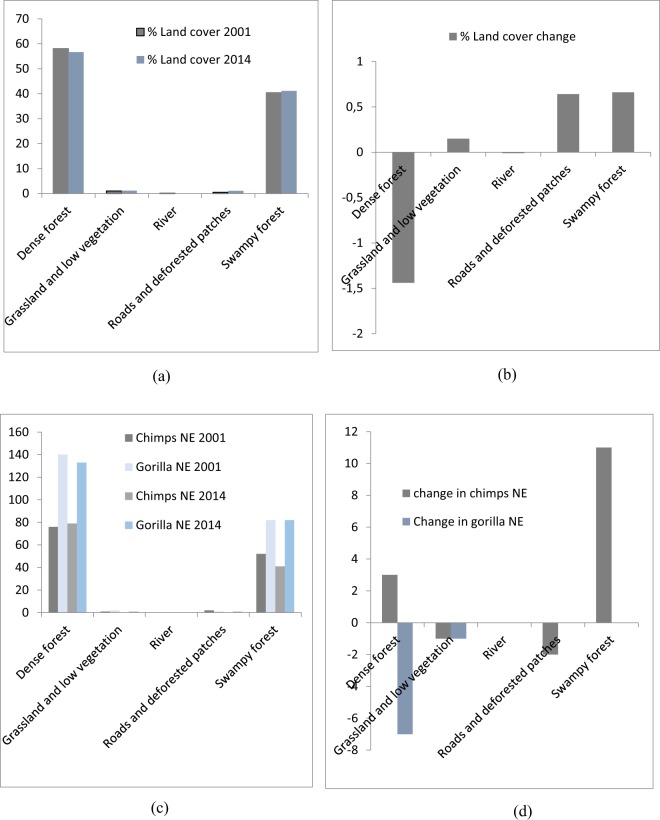
Comparison in land cover and great apes nest encounter at the Lobéké National Park (2001–2014). (**a**) % land cover (2001 and 2014); (**b**) % land cover change area (2014-2001); (**c**) great apes nest encounter (2001 and 2014) within land cover classes; (**d**) change in great apes nest encounter (2014-2001) per land cover class. *NE = Nest Encounter.

**Figure 4:**
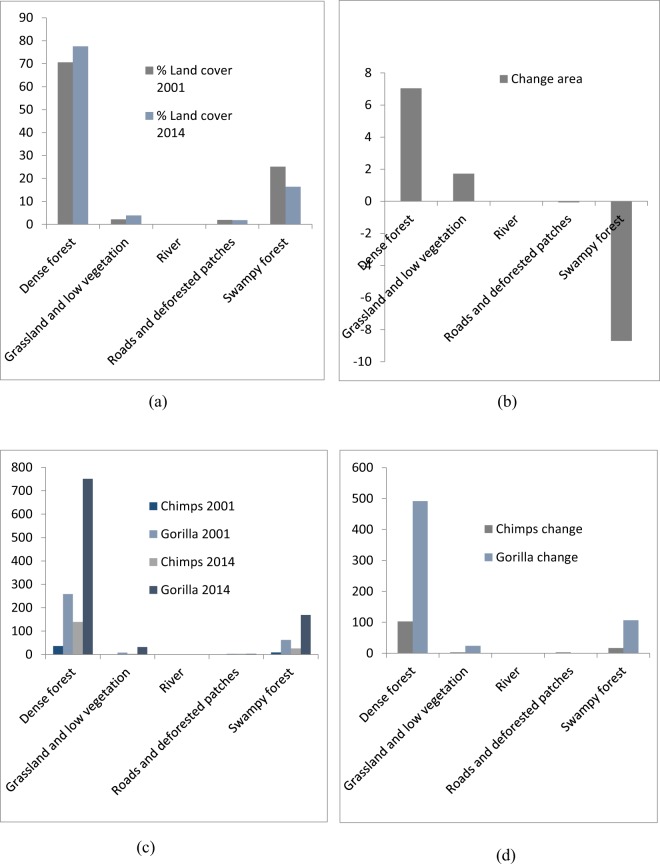
Comparison in land cover and great apes nest encounter at the FMUs (2001-2014). (**a**) % land cover (2001 and 2014); (**b**) % land cover change area (2014-2001); (**c**) great apes nest encounter (2001 and 2014) within each land cover class; (**d**) change in great apes nest encounter (2014-2001) per land cover class. *NE = Nest Encounter.

From the classification as well as great apes distribution data (Figs 1–4 and Tables 3 and 4), it could be observed that, in the Lobéké National Park, dense forests covered a total land cover area of 125129.29 ha in the year 2001, with a total of 76 chimpanzee nests and 140 gorilla nests encountered. Grassland and low vegetated areas covered 2099.1 ha of land with just 1 chimpanzee and 2 gorilla nests encountered. Swampy forests covered a total land cover area of 871034.08 ha with 52 chimpanzee and 82 gorilla nests encountered. Roads and deforested patches covered a total land cover area of 961.89 ha with 2 chimpanzee and 1 gorilla nest encountered. Rivers covered 20.11 ha with no nests encountered. Meanwhile in the same year (2001) in the forest management units, dense forests occupied a total land cover area of 509532.82 ha with a total of 36 chimpanzee and 751 gorilla nests encountered. Grassland and low vegetated areas covered 16007.3 ha of land with 8 gorilla and zero chimpanzee nest encountered. Swampy forests covered a total land cover area of 181149.39 ha with 9 chimpanzee and 62 gorilla nests encountered. Roads and deforested patches covered a total land cover area of 13748.68 ha with 3 gorilla and zero chimpanzee nest encountered. Rivers covered 788.27 ha with no nest encounter observed.

**Table 3:** Chimpanzee and gorilla nest encounter within land cover categories as well as change in nest encounter per land cover change.

Lobéké National Park																		
	2001						2014						Change (2014–2001)					
Land cover data	Land cover area		Chimps		Gorillas		Land cover area		Chimps		Gorillas		Land cover area		Chimps		Gorillas	
Land cover types	Area (ha)	% Area	NE	NER	NE	NER	Area (ha)	% Area	NE	NER	NE	NER	Area (ha)	% Area	NE	NER	NE	NER
Dense forest	125129.29	58.11	76	0.58	140	1.08	122028.14	56.67	79	0.5	133	0.84	−3101.12	−1.44	3	−0.08	−7	−0.24
Grassland and low vegetation	2099.10	0.97	1	0.01	2	0.02	2427.08	1.13	0	0	1	0.01	327.98	0.15	−1	−0.01	−1	−0.01
River	20.11	0.01	0	0	0	0.00	3.01	0	0	0	0	0	−17.11	−0.01	0	0	0	0
Roads and deforested patches	961.89	0.45	2	0.02	1	0.01	2330.69	1.08	0	0	1	0.01	1368.8	0.64	−2	−0.02	0	0
Swampy forest	87103.08	40.45	52	0.4	82	0.63	88524.52	41.11	41	0.26	82	0.52	1421.44	0.66	−11	−0.14	0	−0.11
Total	215313.44		131	1.01	225	1.73	215313.44		120	0.76	217	1.37				−0.25	−8	−0.34

*NE = Nest Encounter, NER = Nest Encounter Rate.

**Table 4:** Chimpanzee and gorilla nest encounter within land cover categories as well as change in nest encounter per land cover change.

Forest Management units (FMUs)																		
Land cover data	2001						2014						Change (2014–2001)					
	Land cover area		Chimps		Gorillas		Land cover area		Chimps		Gorillas		Land cover area		Chimps		Gorillas	
Land cover types	Area (ha)	% Area	NE	NER	NE	NER	Area (ha)	% Area	NE	NER	NE	NER	Area (ha)	% Area	NE	NER	NE	NER
Dense forest	509532.82	70.65	36	0.06	258	0.42	556707.98	77.68	139	0.22	751	1.18	47175.16	7.04	103	0.16	492	0.76
Grassland and low vegetation	16007.3	2.22	0	0	8	0.01	28198.32	3.93	3	0	32	0.05	12191.02	1.72	3	0.005	24	0.04
River	788.27	0.11	0	0	0	0	924.09	0.13	0	0	0	0	135.82	0.02	0	0	0	0
Roads and deforested patches	13748.68	1.91	0	0	3	0	13151.66	1.84	3	0	3	0	−597.02	−0.07	3	0.005	0	0
Swampy forest	181149.39	25.12	9	0.01	62	0.1	117646.11	16.42	26	0.04	169	0.26	−63503.28	−8.7	17	0.03	107	0.17
Total	721226.46		45	0.07	332	0.53	716628.16		171	0.27	955	1.49	−4598.3		126	0.2	623	0.96

*NE = Nest Encounter, NER = Nest Encounter Rate.

Meanwhile in 2014 in the Lobéké National Park, dense forests covered a total land cover area of 122028.14 ha with a total of 79 chimpanzee nests and 133 gorilla nests encountered. Grassland and low vegetated areas covered 2427.08 ha of land with just 1 gorilla and zero chimpanzee nest encountered. Swampy forests covered a total land cover area of 88524.52 ha with 41 chimpanzee and 82 gorilla nests encountered. Roads and deforested patches covered a total land cover area of 2330.69 ha with 1 gorilla and zero chimpanzee nest encountered. Rivers covered 20.11 ha with no nests encountered. Meanwhile in the same year (2014) in the forest management units, dense forests occupied a total land cover area of 556707.98 ha with a total of 139 chimpanzee and 751 gorilla nests encountered. Grassland and low vegetated areas covered 28198.32 ha of land with 32 gorilla and 3 chimpanzee nests encountered. Swampy forests covered a total land cover area of 117646.11 ha with 26 chimpanzee and 169 gorilla nests encountered. Roads and deforested patches covered a total land cover area of 13151.66 ha with 3 gorilla and 3 chimpanzee nest encountered. Rivers covered 924.09 ha with no nest encounter observed.

The change detection results indicate that, in the Lobéké National Park, dense forest have decreased by 3101.12 ha (1.44%), causing a decrease in 3 chimpanzee and 7 gorilla nests. Grassland and low vegetated areas have increased by 327.98 ha (0.15%) leading to a decrease in 1 chimpanzee and 1 gorilla nest. Swampy forests have increased by 1421.44 ha (0.66%) with no change in gorilla nests but a lost in 11 chimpanzee nests. Roads and deforested patches have increased by 1368.8 ha (0.64%) with no change in gorilla nest but a lost in 2 chimpanzee nests. While rivers have decreased by 17.11 ha (0.01%) with no effect on chimpanzee and gorilla nests.

**Figure 5:**
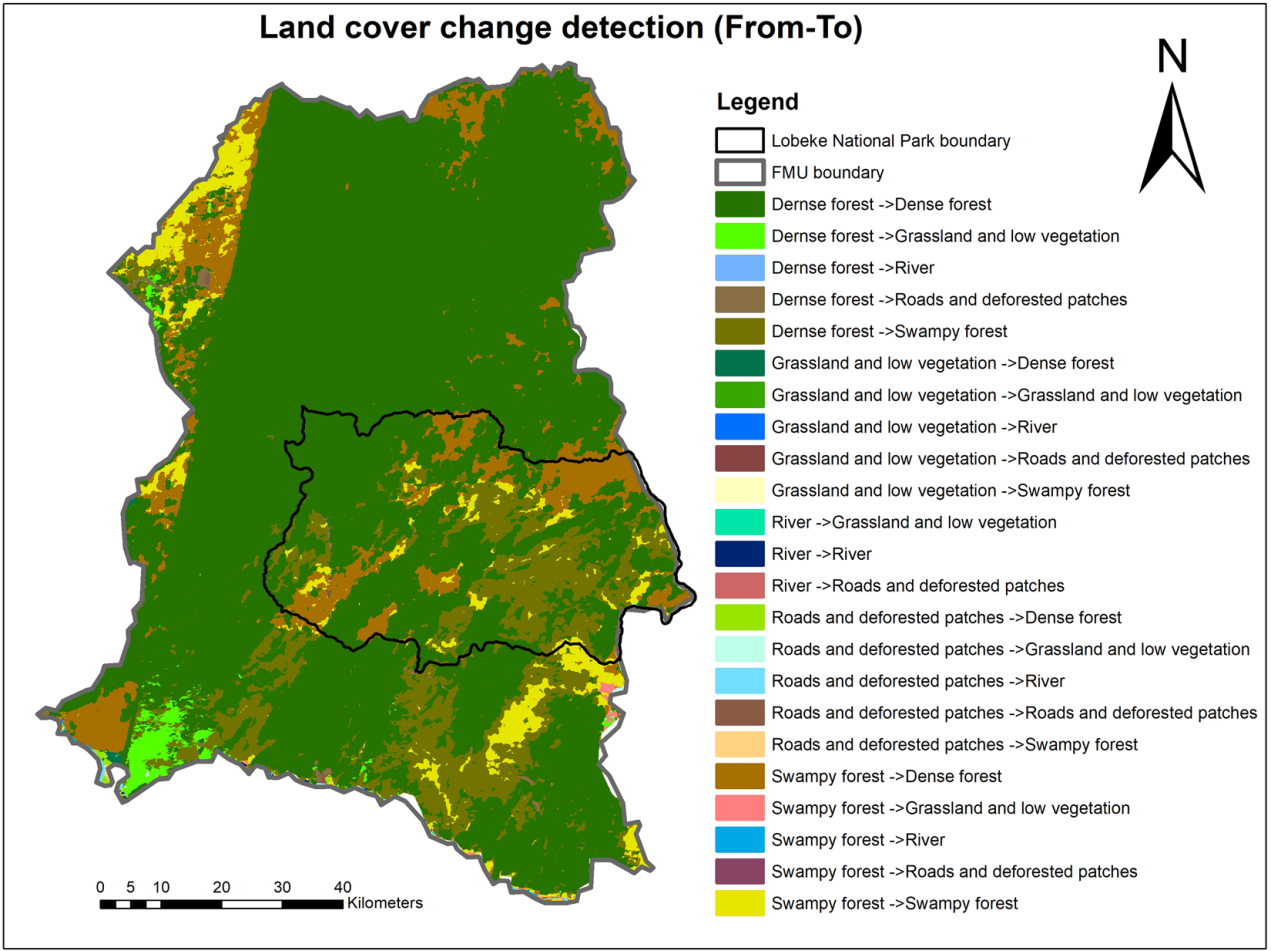
Land cover change detection from one land cover type to another and vice-versa (Map prepared with ArcGIS 10.3.1).

From the land cover classification data in Tables 3 and 4 and Figs 1 and 2, detected changes in land cover were deduced with the criteria “From-To” (i.e. from one land cover type to another and vice versa). Figure 5 and Table 5 illustrate the detected changes, which shows that, at the Lobéké National Park between 2001–2014, approximately 1773.12 ha of the entire land cover area has been converted from dense forest to grassland and low vegetation causing a decrease in one chimpanzee nest but with no change in gorilla nest. About 977.43 ha of land has been converted from dense forest to roads and deforested patches causing a decrease in 2 chimpanzee nests but no change in gorilla nest. About 36038.49 ha of land has changed from dense forest to swampy forest leading to a decrease in 6 chimpanzee nests and gain in 1 gorilla nest. Approximately 1775.79 ha of land has changed from grassland and low vegetation to dense forest leading to an increase in 14 chimpanzee nests and a decrease in 2 gorilla nests. About 244.57 ha of land has changed from grassland and low vegetation to swampy forests causing a decrease in 1 chimpanzee nest and no change in gorilla nests. About 447.58 ha of land has changed from roads and deforested patches to dense forests with a decrease in one gorilla nest but no change in chimpanzee nest. Approximately 42425.11 ha of land has changed from swampy forest to dense forest causing a decrease in 10 chimpanzee and 7 gorilla nests. About 607.89 ha of land has changed from swampy forest to grassland and low vegetation leading to a decrease in 1 chimpanzee nest but with no change in gorilla nest. About 930.95 ha of land has changed from swampy forests to roads and deforested patches with a decrease in 1 chimpanzee nest observed but no change in gorilla nest. All areas that changed from or to rivers recorded no change in chimpanzee and gorilla nests, as observed in Table 5.

**Table 5:** Detected chimpanzee and gorilla nest encounters and encounter rates within detected land cover change areas.

Land cover change (From – To)	Lobéké National Park						FMUs						Full study Area					
	Change area		Chimps change		Gorilla change		Change area		Chimps change		Gorilla change		Change area		Chimps change		Gorilla change	
	Area (ha)	% Area	NE	NER	NE	NER	Area (ha)	% area	NE	NER	NE	NER	Area (ha)	% Area	NE	NER	NE	NER
Dernse forest ->Dense forest	88668.29	41.86	−8	−0.06	7	0.05	408857.87	57.32	68	0.11	361	0.6	497526.16	53.78	60	0.05	368	0.63
Dernse forest - > Grassland and low vegetation	1773.12	0.84	−1	−0.01	—	—	19790.77	2.77	3	0.005	18	0.03	21563.89	2.33	2	—	18	0.03
Dernse forest ->River	0.01	—	—	—	—	—	0.08	—	—	—	0	—	0.09	—	—	—	—	—
Dernse forest ->Roads and deforested patches	977.43	0.46	−2	−0.02	—	—	6798.06	0.95	1	0.002	−2	−0.003	7775.49	0.84	−1	−0.01	−2	—
Dernse forest ->Swampy forest	36038.49	17	−6	−0.05	1	0.01	66772.07	9.36	8	0.013	78	0.13	102810.56	11.11	2	0.03	79	0.13
Grassland and low vegetation ->Dense forest	1775.79	0.84	14	0.11	−2	−0.02	11565.14	1.62	4	0.006	11	0.02	13340.93	1.44	18	0.11	9	—
Grassland and low vegetation ->Grassland and low vegetation	34.33	0.02	—	—	—	—	2481.18	0.35	—	—	2	0.003	2515.51	0.27	—	—	2	—
Grassland and low vegetation ->River	—	—	—	—	—	—	0.02	—	—	—	—	—	0.02	—	—	—	—	—
Grassland and low vegetation ->Roads and deforested patches	25.72	0.01	—	—	—	—	665.37	0.09	—	—	—	—	691.09	0.07	—	—	—	—
Grassland and low vegetation ->Swampy forest	244.57	0.12	−1	−0.01	—	—		0.17	—	—	−1	−0.002	1429.62	0.15	−1	−0.01	−1	—
River ->Grassland and low vegetation	—	—	—	—	—	—	13.01	—	—	—	—	—	13.01	—	—	—	—	—
River ->River	0.02	—	—	—	—	—	354.63	0.05	—	—	—	—	354.66	0.04	—	—	—	—
River ->Roads and deforested patches	—	—	—	—	—	—	2.19	—	—	—	—	—	2.19	—	—	—	—	—
Roads and deforested patches ->Dense forest	447.58	0.21	—	—	−1	−0.01	5022.84	0.7	−1	−0.002	5	0.008	5470.42	0.59	−1	—	4	—
Roads and deforested patches ->Grassland and low vegetation	6.68	—	—	—	—	—	1459.87	0.2	—	—	2	0.003	1466.55	0.16	—	—	2	—
Roads and deforested patches ->River	2.66	—	—	—	—	—	420.88	0.06	—	—	—	—	423.54	0.05	—	—	—	—
Roads and deforested patches ->Roads and deforested patches	182.53	0.09	—	—	1	0.01	2601.6	0.36	1	0.002	−1	−0.002	278.12	0.3	1	—	—	0.01
Roads and deforested patches ->Swampy forest	152.19	0.07	−1	−0.01	—	—	3195.7	0.45	—	—	3	0.005	3347.88	0.36	−1	−0.01	3	—
Swampy forest ->Dense forest	42425.11	20.03	−10	−0.08	−7	−0.05	116107.82	16.28	28	0.05	101	0.16	158532.93	17.14	18	−0.03	94	0.11
Swampy forest ->Grassland and low vegetation	607.89	0.29	−1	−0.01	—	—	3966.94	0.56	—	—	7	0.011	4574.83	0.49	−1	−0.01	7	0.01
Swampy forest ->River	0.32	—	—	—	—	—	10.19	—	—	—	—	—	10.51	—	—	—	—	—
Swampy forest ->Roads and deforested patches	930.95	0.44	−1	−0.01	—	—	2601.65	0.36	—	0.002	1	0.002	3532.6	0.38	—	−0.01	1	—
Swampy forest ->Swampy forest	37521.98	17.71	10	0.08	−7	−0.05	59417.2	8.33	12	0.02	40	0.064	96939.17	10.48	22	0.1	33	0.01
Total	211815.65	100	−7	−0.05	−8	−0.06	713290.13	100	125	2	625	1.002	925105.78	100	118	0.15	617	0.94

*NE = Nest Encounter, NER = Nest Encounter Rate.

Similarly within FMUs, approximately 19790.77 ha of land has changed from dense forest to grassland and low vegetation leading to increase in 3 chimpanzee and 18 gorilla nests. About 6798.06 ha of land has changed from dense forest to roads and deforested patches with an increase in 1 chimpanzee and decrease in 2 gorilla nests. About 66772.07 ha of land has changed from dense forest to swampy forest with an increase in 8 chimpanzee and 78 gorilla nests observed. Approximately 11565.14 ha of land has changed from grassland and low vegetation to dense forests leading to increase in 4 chimpanzee and 11 gorilla nests. About 665.37 ha has changed from grassland and low vegetation to roads and deforested patches with no change in chimpanzee nor gorilla nest observed. About 1185.05 ha of land has changed from grassland and low vegetation to swampy forest with no change in chimpanzee nest but with a decrease in 1 gorilla nest observed. Approximately 5022.84 ha has changed from roads and deforested patches to dense forest with a decrease in 1 chimpanzee nest and an increase in 5 gorilla nest observed. About 1459.87 ha has changed from roads and deforested patches to grassland and low vegetation with no change in chimpanzee nest but with an increase in 2 gorilla nests observed. About 3195.7 ha of land has changed from roads and deforested patches to swampy forests with no change in chimpanzee nest observed but with an increase in 3 gorilla nests observed. Approximately 116107.82 ha of land has changed from swampy forest to dense forest with an increase in 28 chimpanzee and 101 gorilla nests observed. About 3966.94 ha of land has changed from swampy forests to grassland and low vegetation with no change in chimpanzee nest but with an increase in 7 gorilla nests observed.

### Modeling land cover change impacts on species distribution (a 13 year time series analysis)

**Figure 6:**
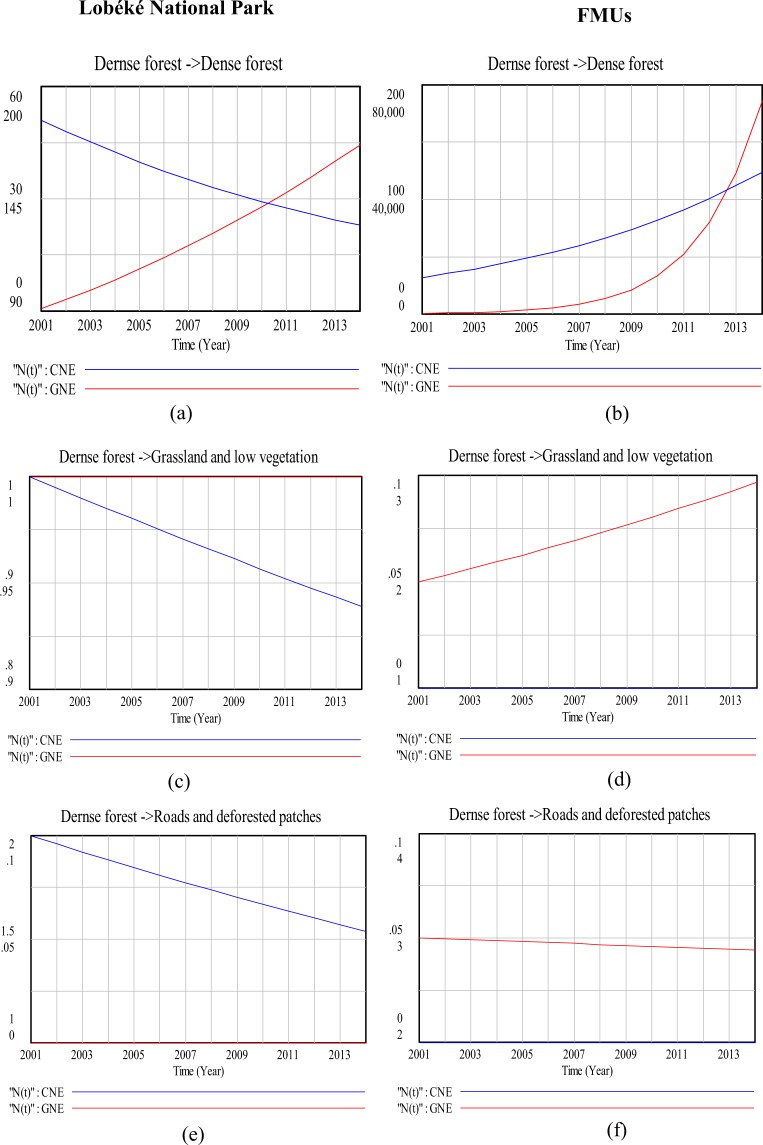
Comparison in land cover change detection impacts on chimpanzee and gorilla nests distributions at the Lobéké National Park and FMUs: (**a**,**b**) are change impacts from dense forest to dense forest; (**c**,**d**) are change impact from dense forest to grassland and low vegetation; (**e**,**f**) are change impacts from dense forest to roads and deforested patches. *CNE = chimpanzee nest encounter and GNE = gorilla nest encounter.

**Figure 7:**
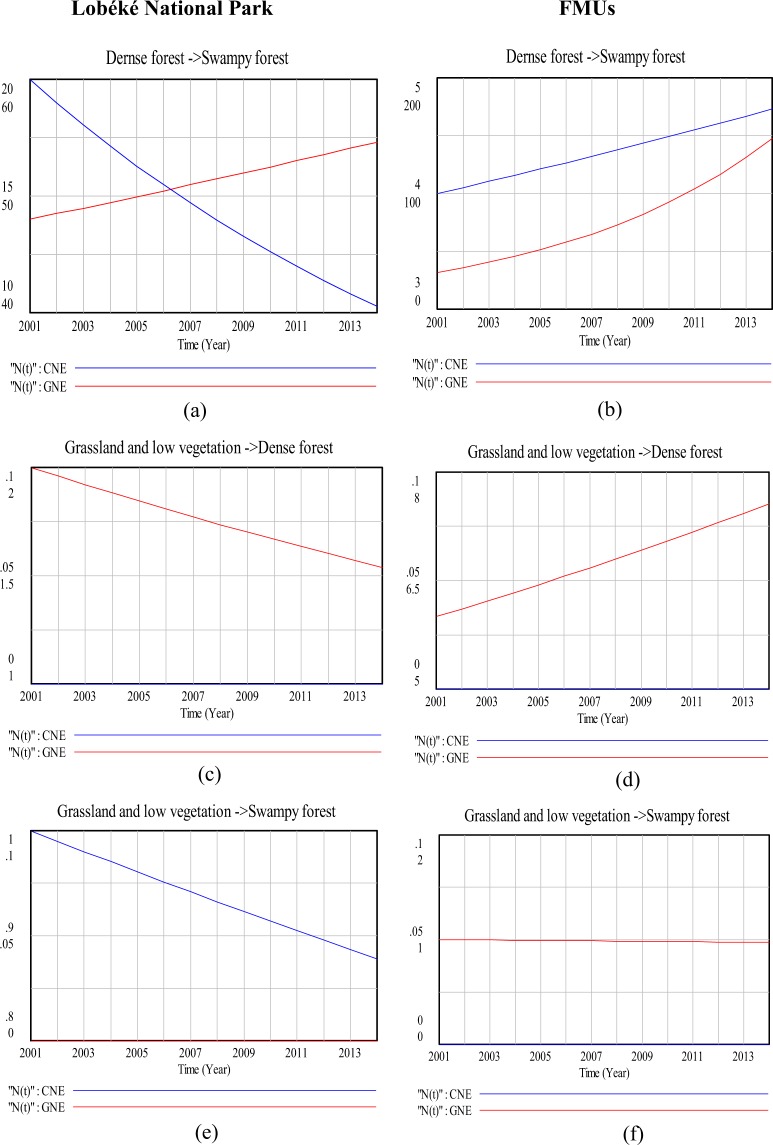
Comparison in land cover change detection impacts on chimpanzee and gorilla nests distributions at the Lobéké National Park and FMUs: (**a**,**b**) are change impacts from dense forests to swampy forests; (**c**,**d**) are change impacts from grassland and low vegetation to dense forests; (**e**,**f**) are change impacts from grassland and low vegetation to swampy forests. *CNE = chimpanzee nest encounter and GNE = gorilla nest encounter.

**Figure 8:**
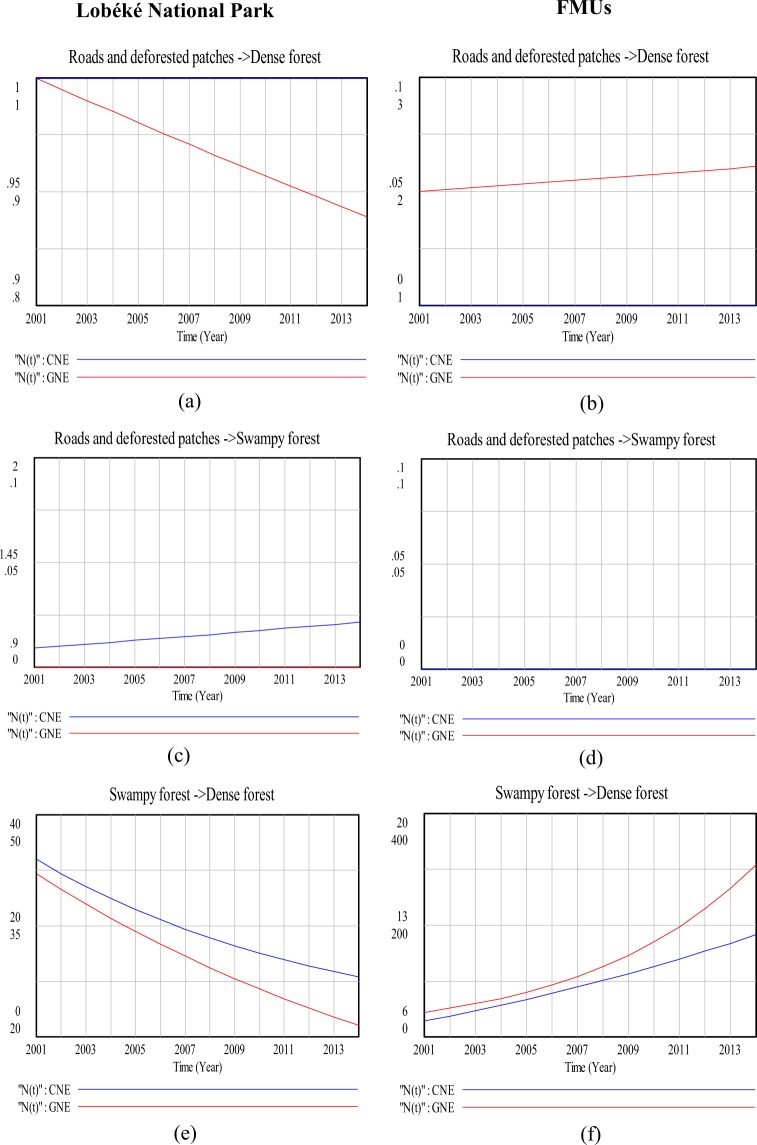
Comparison in land cover change detection impacts on chimpanzee and gorilla nests distributions at the Lobéké National Park and FMUs: (**a**,**b**) are change impacts from roads and deforested patches to dense forests; (**c**,**d**) are change impacts from roads and deforested patches to swampy forest; (**e**,**f**) are change impacts from swampy forests to dense forest. *CNE = chimpanzee nest encounter and GNE = gorilla nest encounter.

**Figure 9:**
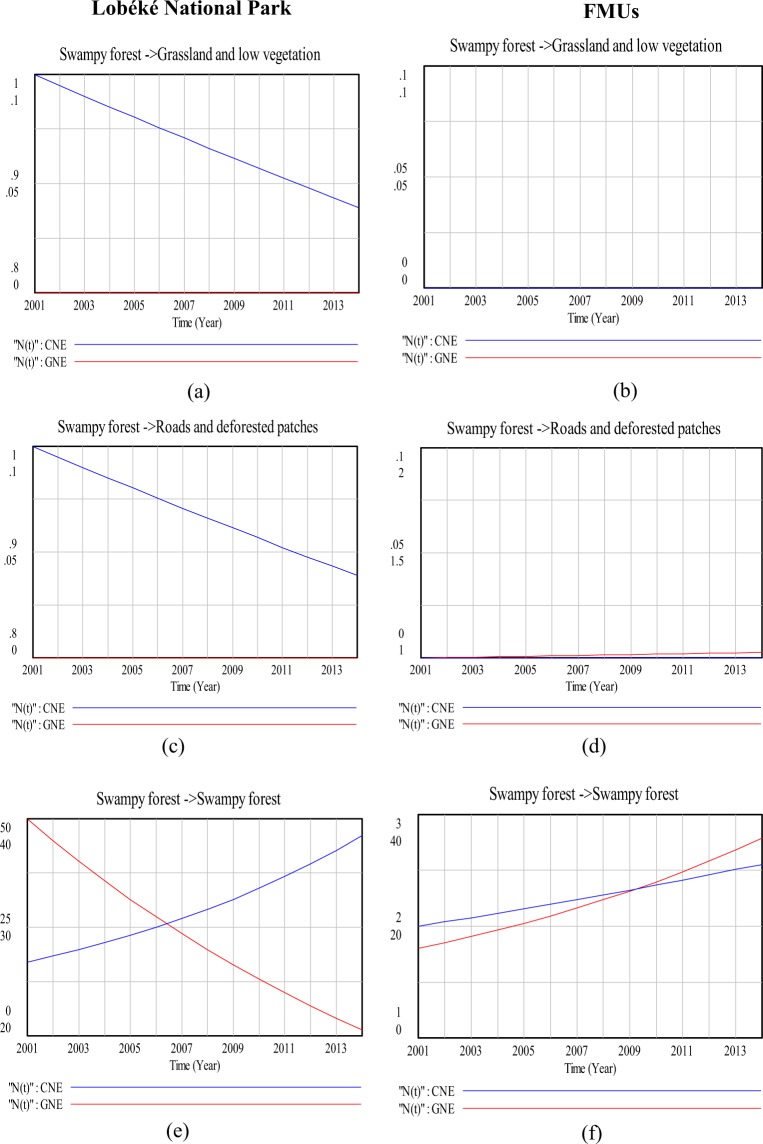
Comparison in land cover change detection impacts on chimpanzee and gorilla nests distributions at the Lobéké National Park and FMUs: (**a**,**b**) are change affects from swampy forests to grassland and low vegetation; (**c**,**d**) are change affects from swampy forests to roads and deforested patches; (**e**,**f**) are change affects from swampy forests to swampy forests. *CNE = chimpanzee nest encounter and GNE = gorilla nest encounter.

**Figure 10:**
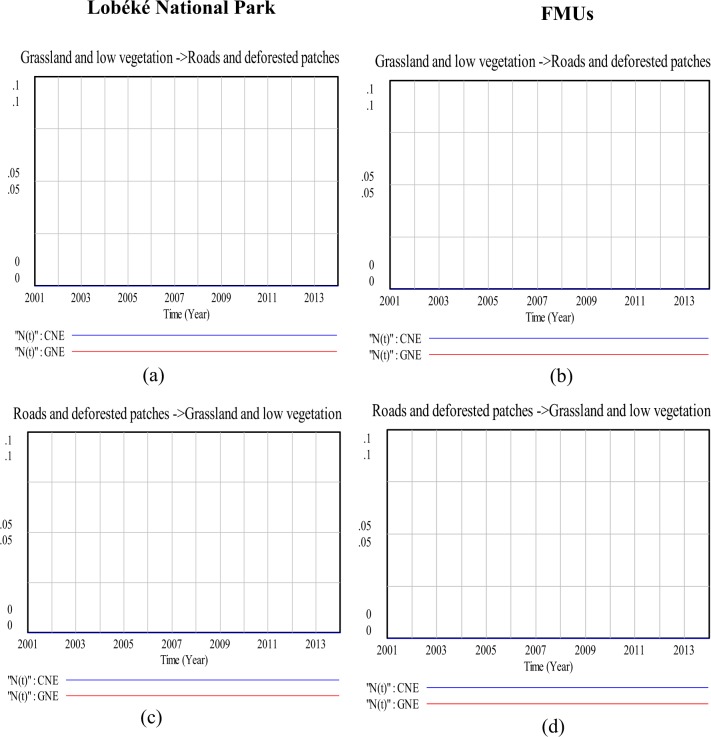
Comparison in land cover change detection impacts on chimpanzee and gorilla nests distributions at the Lobéké National Park and FMUs (**a**,**b**) are change impacts from grassland and low vegetation to roads and deforested patches; (**c**,**d**) are change impacts from roads and deforested patches to grassland and low vegetation. *CNE = chimpanzee nest encounter and GNE = gorilla nest encounter.

With the logistic growth and regression equations (equations 3 and 4) applied in the vensim model, the rate of great apes nest increase or decrease as a result of land cover change impacts was deduced for both the Lobéké National Park and the FMUs (Figs 6–10), taking into consideration the parameters “r”(change in nest encounter rate), “k”(Land cover change area: constituting the impact), “N0” (Initial nest encounters per land cover change for the year 2001) and “N(t)”(final nest encounter by the year 2014 as a result of land cover change impact). Table 5 show the respective data sets applied in the vensim model to obtain the results of Figs 6–10. The data sets were prepared by selecting apes nests within detected changed areas (“From-To) using the selection by attribute and location tool in ArcGIS.

## Discussion

The data analysis emphasized on preparing a land cover classification for the areas of interest (Lobéké National Park and its FMUs) as well as a change detection analysis on the land cover classes, from which, validation was done with a pivot table as well as through comparison with global forest cover data. The analysis further considered the effects of these land cover changes to great apes distribution using nest count data derived in the field through distance sampling techniques with line transects^27–29^. The results obtained through our analysis provides an in-depth knowledge on how changes in land cover affects wildlife species distribution within protected areas, using great apes as a reliable case study.

From our land cover classification and change results, we found out that, large areas of dense and swampy forests were unaffected within the course of 13 years (i.e. 41.86% and 17.1% respectively for the Lobéké National park and 57.32% and 8.3% respectively for the surrounding FMUs). Meanwhile, in the Lobéké National Park, 20.3% area has changed from swampy forest to dense forest and 17% from dense forest back to swampy forest. In the FMUs, 9.36% area has changed from dense forest to swampy forest and 16.28% changed from swampy forests to dense forests. These figures thus showed that, approximately 95% land cover area at the Lobéké National Park has never been logged while 91% land cover area at the FMUs are in tack as well, averaging an unchanged forest cover in the entire study area at approximately 93%. This indicates that, strong and advanced conservation measures are being implemented to protect the forest diversities within the park and its surrounding FMUs^30,31^. Of the remaining 7% changed areas, we observed that, 1.3% land cover area has changed from all other cover types to grasslands in the Lobéké National Park and 4.7% areas changed to grasslands in the FMUs, averaging approximately 3% land cover area converted to grasslands and low vegetation in the entire study area. Similarly, in the Lobéké National Park, 0.9% land cover area has changed to roads and deforested areas with 1.4% similar change observed at the FMUs, averaging a 1.2% total area changed to roads and deforested areas in the entire study region. Meanwhile a 1.2% change from all cover types to forests were observed in the Park with 2.5% observed at the FMUs, averaging a 2% conversion of other cover types to forests in the entire study region.

Within the land cover data, we found out that a large number of chimpanzee and gorilla nests were encountered within dense and swampy forests between both years (2001 and 2014) with gorilla nests completely dominating especially within FMUs. This is because managed forests are particularly valuable to large mammals and other smaller animals when surrounded by protected areas or FMUs^32^ especially in cases where hunting laws are strictly implemented^33^. In addition, large conservation landscapes found within protected areas usually surrounded by community forests, logging concessions or swampy forests maximize the area of habitat available to wildlife^34,35^. The dominance in gorilla distribution as compared to chimpanzees in this regards is attributed to the quality and distribution of their habitats^36–39^ as studies have shown that gorillas frequently occur within protected areas and certified logging concessions largely dominated by swamps than chimpanzees^40^. Their risk of extinction in various protected areas across Africa are quite high following changes in forest cover since these species are more vulnerable to hunting and are slow breeders, consequently requiring large amounts of habitat to accommodate their resource demands^41^.

The effects of changes between the two forest types on great apes were quite different when modeled with Vensim (Figs 7a,b and 8e,f). For example, in the Lobéké National park, we found out that, as dense forests changed to swampy forests, there was a steady decline in chimpanzee nest but with an increase in gorilla nest. For the FMUs, the encounter rates of both species increased between both years. These trends were similar to those already documented from other studies across Cameroon, Gabon and Northern DRC that, chimpanzees occur at low densities within swampy forests whereas gorillas occur at high densities within protected areas and certified logging concessions largely dominated by swampy forests^40^. In changed areas from swampy forests to dense forests, we also found a steady decrease in the nest encounter of both species at the Lobéké National Park between both years, but with a steady increase within FMUs.

Our study also shows that, changes from dense forests and swampy forests to grassland and low vegetation caused a steady decrease in chimpanzee nests at the Lobéké National Park but with increase in gorilla nests within FMUs (Figs 6c,d and 9a,b). These trends were quite predictive as studies have shown that gorillas preferentially use habitat types characterized by abundant herbaceous vegetation often composed of mature secondary forests^42,43^ while chimpanzees prefer undisturbed areas^39,44–48^. Evidence from related studies have also shown that, changes in wildlife abundance over time is best attributed to shifts in forest structure through reduction in canopy cover which generally creates a surge of new growth of grasses, shrubs, and herbs on the forest floor^49,50^. We also found out that, changes from swampy forests and dense forests to roads and deforested patches caused a steady decline in chimpanzee nests within both the Park and the FMUs but with a static state in gorilla nests. The conversion of forested areas to roads negatively affects wildlife species in general. The creation and construction of roads or pavements within forested areas limits the physical movement of most wildlife species^51^ and as well pave way for increase hunting and deforestation^52,53^. Hence, the steady decline in chimpanzee nests within the said changed areas contributes to the already documented evidence that, chimpanzees densities are much more lower in logged areas (deforested areas) and areas frequently disturbed by humans than undisturbed areas^39,44–48^.

Some changes in land cover type had little or no impact on great apes distribution. For examples, the conversion of various land cover changed areas from roads and deforested areas to grassland and low vegetation, and vice versa proofed an insignificant impact on great apes distribution (Figs 10a,b and 10c,d). The conversions of various land cover types to rivers and vice versa were equally insignificant in influencing great apes distribution. This is because very little or no amount of land cover were changed between the said conversions.

## Conclusion

With a 93% intact forest cover observed in the entire study area, our study could still demonstrate various change effects of land cover on great apes though limited. The study could also show that, change effects vary differently within National parks and forest management units. Given that limited studies have been carried out to document such effects, we suggest that further research in largely logged protected areas and FMUs be done across Africa, and as well consider other large mammal or primate species with long term data availability. This will extend our findings to a much broader extent and as well, provide more light on understanding the effects of land cover change on species distribution through time series analysis. Such studies will provide baseline information required to improve strategic planning on long-term sustainability of protected areas and logging concessions, especially in areas were management considerations are weak.

## Materials and Methods

### The Study Area

The Lobéké National Park and its surrounding FMUs are located in South-East Cameroon around the Moloundou subdivision of the Eastern region of the country. The study area that lies between latitudes 2° 05′ to 2° 30′ N and longitudes 15° 33′ to 16° 11′ E is bounded on the East by the Sanaga river which serves as Cameroon’s international border with Central African Republic and the Republic of the Congo. The park covers an area of 217334 ha with an altitude ranging from 300 m (980 ft.) to 750 m (2460 ft.) above sea level. The surrounding FMUs comprise a total area of 717550 ha (i.e. ZIC 31 or UFA 10–064 with an area of 115 917 ha, ZIC 30 or UFA 10–012 with an area of 74 504 ha, ZIC 29 or UFA 10–009 and 10-010 with an area of 177 317 ha, ZIC 28 or UFA 10–00782 with an area 144 ha, ZICGC1 or UFA 10–007 with an area of 55 309 ha, ZICGC 2 or UFA 10–013 with an area of 128 541 ha and la ZICGC 3 or UFA 10–064 with an area of 83 818 ha). The study area is also part of a trans-boundary regional protected area network that includes two other National Parks: Nouabale-Ndoki National Park, NNNP (Congo-Brazzaville) and Dzanga-Ndoki (Central African Republic) all funded by the Central African Forest Commission (COMIFAC) and managed by WWF, GIZ and WCS Cameroon.

Lobéké is predominantly a semi-evergreen forest characterized by dominant species of plants such as; Sterculiaceae (*Triplochiton pterygota*), *Ceiba pentandra* and *Terminalia superba*. The fauna is characterized by various species of wildlife such as;elephants, western lowland gorillas, chimpanzees, leopards and a few species of ungulates. In addition to mammals, the park also inhabits numerous species of reptiles as well as butterflies, birds and fishes.

### Image acquisition and pre-processing

Landsat 7 ETM + images of South-East Cameroon with acquisition dates of February 2001 and February 2014 were downloaded from usgs.earthexplorer.gov at path/row 182/59 with 70% cloud cover. The downloaded images were geo-referenced in ArcGIS 10.3.1at a coordinate reference system of WGS 84 UTM zone 33N in order to validate accuracy in the country of origin (Cameroon). Multispectral band stacking was done in Erdas Imagine 2014 for proper visualization. In order to improve image quality for proper land cover classification and vegetation indices quantification, atmospheric correction of both images was done using Landsat 7 surface reflectance in Erdas Imagine 2014 to remove atmospheric gases and aerosols. The objective of atmospheric correction was to convert remotely sensed DN (digital numbers) to ground surface reflectance. This image based correction method was based on the gain and bias and the maximum and minimal radiance value header file produced during image acquisitions^54^. Equations 1 and 2 show the algorithms used in the atmospheric correction process.

1 $${{\rm{L}}}_{{\rm{\lambda }}\mathrm{sensor}}={{\rm{Gain}}}_{\lambda }\,\ast \,{{\rm{DN}}}_{\lambda }+{{\rm{Bias}}}_{\lambda }$$

2 $${{\rm{L}}}_{{\rm{\lambda }}\mathrm{sensor}}=({{\rm{L}}}_{{\rm{\lambda }}\max }-{{\rm{L}}}_{{\rm{\lambda }}\min })/{{\rm{L}}}_{{\rm{\lambda }}\mathrm{range}}\,\ast \,{\rm{DN}}\lambda +{{\rm{L}}}_{{\rm{\lambda }}\min }$$

From the atmospheric corrected images, an area of interest (AOI) for both years, comprising the Lobéké National Park was extracted for further processing.

### Image classification and change detection

Image classification uses the reflectance statistics for individual pixels, while change detection identifies and distinguishes the different land use and land cover types with respect to change in time^55^. Several techniques of change detection analyses have been developed by remote sensing scientists over the past decades with 11 of them commonly used^56^. In this paper, we identified supervised classification to be the best approach to classify and perform change detection on our area of interest since little or no information is available on the land cover classification of this area. Supervised classification was performed for both the years 2001 and 2014 in the entire study area (i.e. Lobeke National Park plus its surrounding FMUs), in order to extract quantitative information on the different land cover classes from the selected area of study or interest^57^. In this processing technique, training and validation files were produced with assigned land cover classes selected from the image pixels in ArcGIS 10.3.1. Signature files were created from the training data and a maximum likelihood classification performed. The classified results were further validated using the validation data and a confusion matrix produced in Microsoft excel in order to assess classification accuracy^23,25^. Classification accuracies were also validated through comparison of forest loss-forest gain calculations with global forest cover data. In ArcGIS 10.2.2, the classified results in raster formats were further converted to vector shape files and a map intersect created with a sub field “From-TO” in order to perform change detection. The areas of the classified land cover categories as well as their detected changes were further calculated in hectares using spatial statistics with the geometry tool (Tables 3–5). An extract of these calculations was done separately for the Lobeke National Park and the corresponding FMUs. This enables a comparison in change effects between the National Park and its FMUs. This method involved observing and comparing two multi-temporal images acquired of the same geographical area at different anniversary dates (2001 and 2014) in order to map and analyze spatial patterns of change^58^.

### Survey design and species distribution data collection

Chimpanzee and Gorilla distribution data for the years 2001 and 2014 were collected at the Lobéké National Park and its surrounding FMUs by a team of WWF biomonitoring and survey experts, using the method of distance sampling with line transects^27–29^. The implemented technique followed a standardize protocol: IUCN best practices for the survey of great apes^59^. The data collection team comprised of eight teams with eight field assistants per team. In each team, there was a GPS and topofil operator (in charge of making ground observations and measuring transect distances), a script or data entry assistant (charged with recording all Apes observations both on ground and tree canopies) and a decameter operator (charged with recording all human activities and measuring perpendicular distances for every observation made). These task separations minimized the effects of double observation and counting during transect walks.

**Figure 11:**
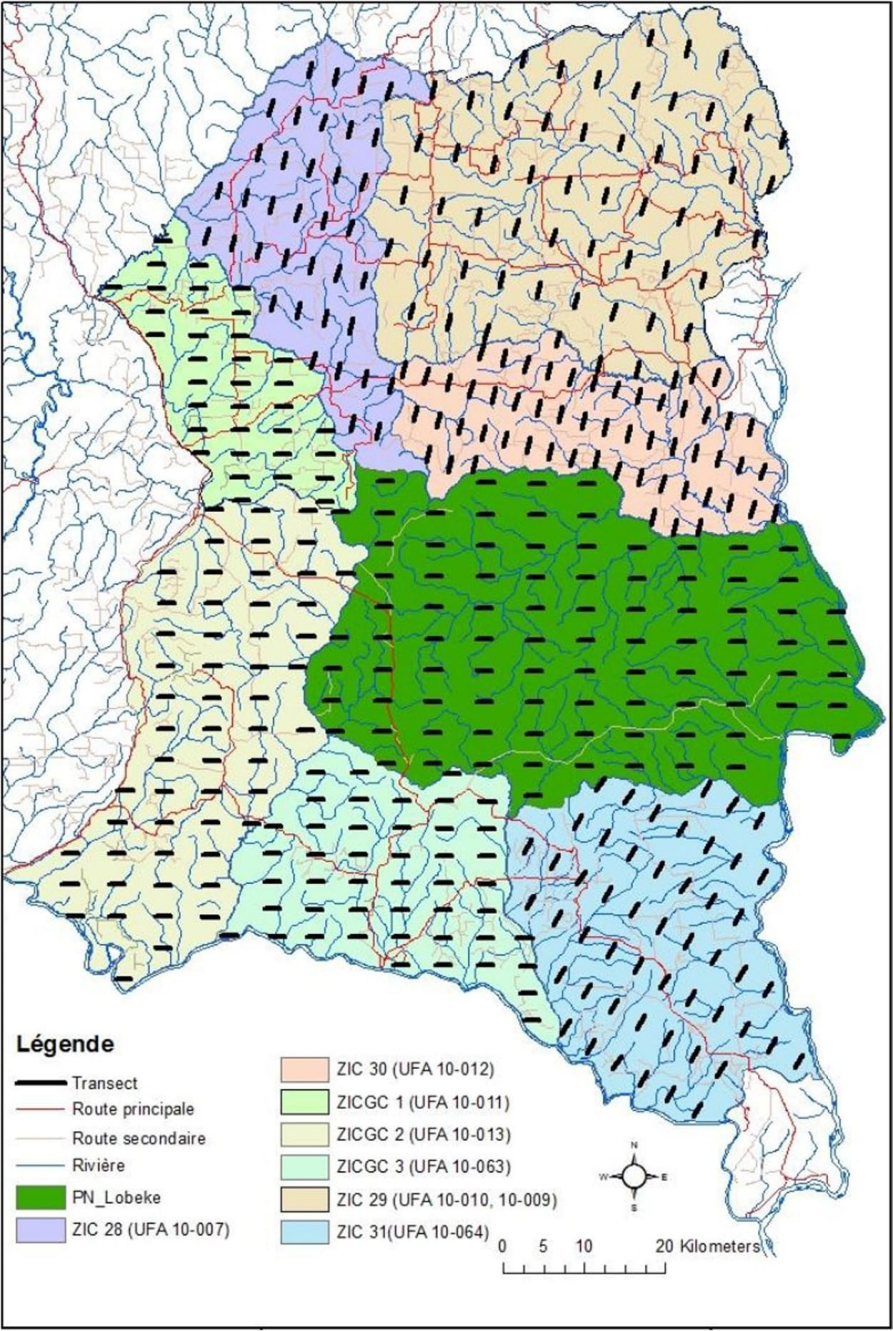
Sampling map showing the location of transects in the study area (Map prepared with ArcGIS 10.3.1).

**Figure 12:**
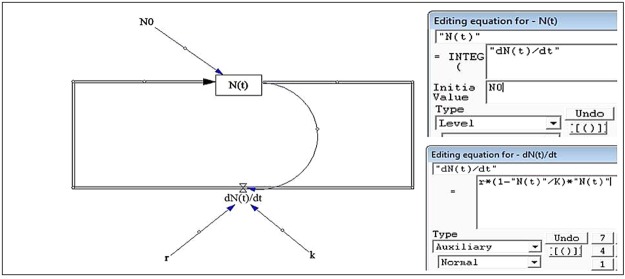
Vensim model applied to simulate the rate of change in great apes distribution per time, impacted by rate of change in land cover (Model developed with Vensim PLE 7.2).

During the 2014 survey period therefore, a total of 797 km distance transect was covered, of which 158 km effort was realized at the Lobéké National Park and 639 km realized at the surrounding FMUs. During the 2001 survey period, 130 km transect effort was covered at the Lobéké National Park while 624 km transect efforts were covered in the surrounding FMUs at extended survey dates of 2003, 2004 and 2005. Stratification of the landscapes for data collection was done according to the individual forest management units (UFA 10–064/ZIC 31, UFA 10–012 /ZIC 30, UFA 10-009 et 10-010/ZIC 29, UFA 10-007/ZIC 28, UFA 10–011/ZICGC 1, UFA 10–013/ZICGC 2, UFA 10–063/ZICGC 3) and the Lobéké National Park (Figs 11 and 12). The determination of the macro-zone sounding effort was made by combination of national standards for wildlife inventories in forest areas (decree N ° 0221/MINFOF of May 02, 2006) and international statistical principles based on the rate meeting the presence indices of the target species (great ape nests). During analysis, we combined all forest management units together in order to provide a general statistical over view of FMUs effects as a whole, an as such differentiated the said analysis from the National Park for comparison purposes (Tables 3–5)

During transect efforts, chimpanzee and gorilla nests as well as other observations (e.g. vocalization, faeces, footprints, tracks etc.) were recorded. Nests were counted individually for each ape species in order to avoid over estimation that could arise through group counts. For each nest or group of nests observed, perpendicular distances from line transects were measured while the nest age or decay stage (i.e. whether fresh or old), height, type (i.e. whether gorilla or chimpanzees) and number were recorded. Each nest was classified as definitely gorilla or chimpanzee through verified signs of faeces, odour or hair. In addition, all nests observed on the ground as well as associated arboreal nests of the same age class as the nests on the ground were attributed to gorillas, considering that no signs of ground nesting has been documented for the chimpanzees of this study region^39^.

### Data Analysis

A point pattern analysis of chimpanzee and gorilla nests was done within the classified land cover categories of both years in ArcGIS 10.3.1. The analysis combined both fresh and old nests since they both represented similar years of study. The aim of this analysis was to statistically compare the encounter rates of chimpanzee and gorilla nests observed within each land cover type in both years and as well calculate the changes in nest encounter observed per land cover change (Tables 3–5). During analysis, data filtering was done in ArcGIS in order to eliminate repeated records of nests that were recorded at different rounds of transect at similar GPS points. The reason was to minimize double counting for a more reliable change analysis. Nest encounter rates were deduced as number of nests encountered in a given land cover area per distance transect. On the detected land cover changes (“From-To”), a selection by attribute and location was done on great apes nests for both years and a change in nest encounter rates of both species deduced per land cover change. The change analysis were done separately for the National Park and the surrounding FMUs (Figs 1, 2 and 5). The reason was to compare the effects of land cover change on nest encounter between the Park and the FMUs.

### Modeling land cover change impacts on Great Apes distribution

In order to predict the rate of change in chimpanzee and gorilla distribution and occurrence at a time interval of 13 years (2001–2014) influenced by detected changes in land cover types (“From-To”), a logistic growth and regression analysis was selected for modeling in Vensim PLE 7.2 (Figure 12). The Vensim PLE application tool and software is very interactive in tracing behaviors through casual links in the form of a model structure thereby automating reality checks (quality control experiments)^60^. In ecological processes, environmental parameters serve as factors that influence species change when modeling equations are applied and simulated in the software. In this context, land cover change variables were applied in the model to depict impacts on chimpanzee and gorilla distribution within the course of 13 years. The following logistic growth equations were applied in the model:

3 $$\frac{{dN}({t})}{{dt}}={r}\cdot (1-\frac{{N}({t})}{{K}})\cdot {N}({t})$$

Where *r* is the rate of change (increase or decrease) in great apes nest encounter, *K* is the carrying capacity of the environment (detected land cover change areas), and

4 $${N}({t})=\frac{{K}}{{A}\cdot {{e}}^{-{rt}}+1}$$

Where A (constant) $=\frac{{\rm{K}}}{{{\rm{N}}}_{0}}-1$ and N_0_ is the initial Great Ape nest encounter at time t_1_ (2001).

### Ethics Statement

The data collection methods for this study were strictly non-invasive and were approved by the Ethical Board of the WWF Cameroon country office. As such, the data were collected in accordance with Cameroon’s laws and regulations governing animal research. WWF Cameroon works in partnership with the Ministry of forestry and Wildlife in Cameroon and as such, has all the necessary permission and MOUs (memorandum of understandings) for data collection within protected areas in Cameroon. As part of the corresponding Authors Masters research work, field data were obtained from the IUCN Apes database at the Max Planck Institute for Evolutionary Anthropology under approval from the WWF Cameroon country office. The available datasets are strictly not for sharing according to the WWF guides and regulations for data availability. Data are however available from the Author upon permission from the data providers (WWF Cameroon country office) or can be obtained directly from the A.P.E.S database (http://apes.eva.mpg.de/).
